# penalizedclr: an R package for penalized conditional logistic regression for integration of multiple omics layers

**DOI:** 10.1186/s12859-024-05850-2

**Published:** 2024-06-27

**Authors:** Vera Djordjilović, Erica Ponzi, Therese Haugdahl Nøst, Magne Thoresen

**Affiliations:** 1 Department of Economics, Ca’ Foscari University of Venice, Venice, Italy; 2 Department of Biostatistics, University of Oslo, Oslo, Norway; 3 Department of Public Health and Nursing, Norwegian University of Science and Technology, Trondheim, Norway; 4 Department of Community Medicine, Faculty of Health Sciences, The Arctic University of Norway, Tromsø, Norway

**Keywords:** Case–control studies, Conditional logistic regression, Multiple blocks of predictors/features, Stability selection

## Abstract

**Background:**

The matched case–control design, up until recently mostly pertinent to epidemiological studies, is becoming customary in biomedical applications as well. For instance, in omics studies, it is quite common to compare cancer and healthy tissue from the same patient. Furthermore, researchers today routinely collect data from various and variable sources that they wish to relate to the case–control status. This highlights the need to develop and implement statistical methods that can take these tendencies into account.

**Results:**

We present an R package penalizedclr, that provides an implementation of the penalized conditional logistic regression model for analyzing matched case–control studies. It allows for different penalties for different blocks of covariates, and it is therefore particularly useful in the presence of multi-source omics data. Both L1 and L2 penalties are implemented. Additionally, the package implements stability selection for variable selection in the considered regression model.

**Conclusions:**

The proposed method fills a gap in the available software for fitting high-dimensional conditional logistic regression models accounting for the matched design and block structure of predictors/features. The output consists of a set of selected variables that are significantly associated with case–control status. These variables can then be investigated in terms of functional interpretation or validation in further, more targeted studies.

## Background

The matched case–control design is widely employed in biomedical studies, since matching on potentially confounding variables can significantly improve efficiency and statistical power, while mitigating the effect of potential confounders. This design has become popular in studies involving high-throughput assays, leading researchers to propose novel methods for the analysis of high-dimensional matched data, also with the aim of feature or variable selection [12]. As many of these ignore the study design and apply methods not designed for the matched design, this strategy can lead to sub-optimal results [see for instance 2, 18] and potentially missing some important associations. A classical method for taking into account the matched design is offered by conditional logistic regression, either applied to each variable individually or applied to all variables jointly in a multiple regression model [see for instance 2] which is the approach we consider here.

Studies containing several types of high-dimensional measurements for each individual – for instance, DNA methylation, copy number variation and mRNA expression – are becoming increasingly common. Integrating such heterogeneous data layers poses an additional challenge to variable selection, as the optimal penalty parameters can vary across different layers. An intuitively simple solution is to generalize a well-investigated method of penalized conditional logistic regression to allow for different penalties for different data layers. This approach can be particularly useful when the proportions of relevant variables are expected to vary across layers.

The method proposed here is similar in spirit to the popular IPF-lasso [3] and IPFStructPenalty [19] which also consider blocks of covariates. With respect to these packages, in penalizedclr, the emphasis is on variable selection, so that the package also includes a function for performing stability selection in an automatic way, see below. This is different from IPF-lasso and IPFStructPenalty that can be used for both prediction and variable selection. This difference stems from the fact that in conditional logistic regression models, intercept terms are treated as nuisance, rendering predictions for new observations impossible. In view of this, in the context of multiomics data, this method is designed to address the initial challenge of selecting promising biomarker candidates.

Table 1 shows R packages that include functions for estimating penalized logistic regression models. As can be seen from this overview, none of the available packages were designed to take into account both matching and blocks of covariates. The R package penalizedclr is intended to fill this gap.

**Table 1:** Overview of methods and associated R packages for estimating penalized logistic regression models in R

Implementation	Multiple penalties	Matching
clogitlasso [1]	No	Yes
ipflasso [3]	Yes	No
penalized [5]	No	Yes
glmnet [4]	Yes	No
IPFStructPenalty [20]	Yes	No
penalizedclr	Yes	Yes

Results of variable selection procedures in high dimensional settings are known to suffer from limited replicability. To address this issue, our package provides an implementation of stability selection, a general method in which results of the selection procedure are aggregated over different data subsamples [14]. To develop good prediction algorithms useful from a diagnostic and clinical perspective, a biological interpretation of the selected candidates would be conducted and they should be further investigated in a prospective study.

## Implementation

penalizedclr is implemented in R and available from CRAN. A development version is also available from github https://github.com/veradjordjilovic/penalizedclr.

In what follows, we describe the two main functions of the package, penalized.clr, estimating a penalized **c**onditional **l**ogistic **r**egression model allowing for different penalties for different blocks of covariates, and stable.clr.g performing stability selection of variables in the penalized conditional regression model. We then discuss other important aspects of the implementation, such as the choice of the penalization parameters and computation time.

### penalized.clr function

This is a wrapper function for the penalized function of the well-established R package of the same name [5, 6]. A routine for conditional logistic regression is not directly available in penalized, but we exploit the fact that the likelihood of a conditional logistic regression model is the same as that of a Cox model with a specific data structure. In the input, we need to specify the response vector, the stratum membership of each observation, i.e. in case of 1:1 matching, the id of the case–control pair the observation belongs to; the overall matrix of covariates to be penalized, the sizes of the blocks of covariates and the ($L_1$) penalties to be applied to each block. The output is a list including the estimated regression coefficients, along with other useful information regarding the fitted model. It should be stressed, that the vector of penalties has no default value and thus needs to be specified by the user.

### stable.clr.g function

To increase the replicability of research findings – in this case selected variables – we aim to select variables that are robust to small perturbations in the data. To this end, we have implemented stability selection [14] in the function stable.clr.g. Here, most of the required input arguments are the same as in penalized.clr, with the argument lambda.list replacing lambda. The argument lambda.list consists of vectors of $L_1$ penalties to be applied to each penalized block of covariates. Each vector has length equal to the number of blocks. For advice and considerations regarding how to specify lambda.list in practice, we refer to data applications in Sections "The NOWAC lung cancer dataset" and "The TCGA lung adenocarcinoma dataset" and Section "Choice of the tuning parameters" in Appendix. For each vector, 2*B* random subsamples of $\lfloor n/2 \rfloor$ (out of the total of *n*) matched pairs are taken and a penalized model is estimated ($B = 100$ by default). The factor 2 in 2*B* is due to a variant of stability selection that includes complementary pairs of subsamples [17]. For each variable and vector of penalties, a selection probability is estimated as the proportion of fitted models in which the associated coefficient estimate is different from zero. Finally, the estimate of the selection probability of a variable is obtained by taking the maximum selection probability over all considered penalty vectors. The user can then select the variables whose estimated selection probability is above a desired threshold, typically in the range $0.55 - 0.9$.

### Data adaptive choice of penalty parameters

The user needs to specify penalties to be applied in the main functions. In general, choosing the appropriate amount of penalization is challenging, and even more so in the presence of multiple blocks of predictors with different penalties. Let $\varvec{\lambda } = (\lambda _1, \lambda _2, \ldots , \lambda _P)$ represent a vector of $L_1$ penalties, where $\lambda _i$ is the penalty applied to the $i-$th block, and *P* is the number of blocks. In principle, the optimal value can be found by performing a grid search over a *P*-dimensional grid. However, this approach is computationally prohibitive, and less computationally demanding alternatives are typically considered. For instance, in [20] the authors propose a stochastic search over a grid. We follow a different strategy and combine a grid search for a scalar parameter with a heuristic data adaptive strategy as follows. The problem of setting $\varvec{\lambda }$ can be decomposed into two subproblems to be solved independently, as we can write $\varvec{\lambda } = \lambda (1, \lambda _2/\lambda , \ldots , \lambda _P/\lambda )$, where $\lambda$ can be viewed as the overall level of penalization, while the vector $(1, \lambda _2/\lambda , \ldots , \lambda _P/\lambda )$ represents the relative penalties with respect to the first block. In analogy with ipflasso, we refer to this vector as the vector of penalty factors. Our package offers two functions: default.pf that performs a heuristic search for the data adaptive vector of penalty factors (see below), and find.default.lambda that, given a vector of penalty factors, finds $\lambda$ that maximizies the cross-validated conditional log-likelihood, see Section "Choice of the tuning parameters" in Appendix for further details.

To find a data adaptive vector of penalty factors, we follow the heuristic approach of [16]. In this extension of the original IPF-lasso method, a tentative conditional logistic regression model is fitted to all covariates, and for each block, the (relative) penalty is set to be inversely proportional to the mean of the estimated coefficients pertaining to that block. In this way, a block with larger estimated coefficients will have a lower penalty, and vice-versa. This step can be performed for each block separately, i.e. by fitting *P* tentative models, or jointly with all blocks included within a single model, see argument type.step1. Once a vector of penalty factors is obtained in this way, we can call find.default.lambda to find the value of $\lambda$ determining the overall extent of penalization. For more details, we refer to [16] and the penalizedclr package documentation.

### Elastic net penalty

The main focus of the package is on $L_1$ or lasso penalty which, resulting in sparse estimated models, is appropriate for variable selection. Nevertheless, it is well-known that with $L_1$ penalty, the presence of highly correlated variables can have a negative impact on selection stability [11]. Adding a small $L_2$ or ridge penalty can alleviate this issue: our implementation offers this possibility by including the mixing parameter alpha, see package documentation and Section "Choice of the tuning parameters" in Appendix for details.

### Computation time

The computational cost of estimating a penalized conditional logistic model with a given vector of penalties equals the cost of estimating a penalized Cox model. The time consuming part of the analysis is stability selection, which requires fitting 2*Bs* models, where *s* is the number of the vectors of penalties in lambda.list. Fortunately, stability selection is highly amenable to parallelization, which greatly reduces computation time especially when using a cluster of computers (see argument parallel of function stable.clr.g).

## Results

### Simulation study

We illustrate the proposed method with a small simulation study. This simulation study is by no means meant to be exhaustive since many different simulation settings can be envisioned. The main purpose of this study is to illustrate some of the numerous factors that influence performance of the proposed method in real applications. The R code files for reproducing the results reported here are available on github https://github.com/veradjordjilovic/Simulations_penalizedclr.

We considered six different settings described in Table 2, where $p_i$ and $a_i$ denote the dimension and the number of active variables in block *i*, respectively, while $\beta _i$ is the coefficient of an active variable in block *i*, $i=1,2$. Common for all settings is the number of blocks (2), the number of matched pairs (200), the total number of covariates (100) and the total number of active variables (20).

For each setting, we generated 100 datasets, to which we applied a variable selection procedure based on conditional logistic regression as follows. First, we computed data adaptive penalties, as described in Section "Data adaptive choice of penalty parameters". Next, we ran stability selection with $B=50$ on penalized conditional logistic regression with these penalties (Sect. "stable.clr.g function") and a default $\alpha =1$. Finally, covariates with selection probability exceeding 0.55 were selected.

We evaluated performance by estimating power, defined as the proportion of active variables identified by our procedure, and false discovery rate (FDR), defined as the proportion of false discoveries among all discoveries; in this case, the proportion of inactive variables among the selected variables. Power and FDR were averaged over 100 datasets.

We compared our approach to two approaches that in practice could also be considered and applied in this context. The first one is IPF-Lasso [3] with an unconditional logistic regression model, and the second one is the conditional logistic regression with a single block of covariates. The former method, implemented in the package of the same name, takes into account the presence of different types of covariates, but ignores matching, the latter, implemented in the R package clogitL1 [15] fits the conditional logistic regression model but ignores the block structure of covariates.

Results are shown in Table 2. We fit clogitL1 only in settings 1 and 4, since in the unconditional model all settings but setting 4 are equivalent, differing only in the position of active variables. We first notice that, in general, the two competing approaches select more variables then our method. In particular, the conditional model achieves reasonable power: 0.85 and 0.64, respectively, with quite high FDR: slightly below 50%. IPF-Lasso in settings 3, 4, 5 and 6 identifies almost all active variables. However, there are also many false positives (FDR in the range of 0.57$-$0.79). Note that this is expected since variable selection with IPF-Lasso and clogitL1 was performed based on a single model fit. Coupling stability selection with these methods is expected to decrease the number of selected null variables. In settings 1 and 2, the number of selected variables with IPF-Lasso is close to that of our approach, with the latter showing a slightly better performance in terms of power and FDR. For our approach, the power is lowest in settings 1, 4 and 6, in which either there is no (considerable) difference in the proportion of active variables in the two blocks (1 and 6) or the signal in one of the blocks is relatively weak. On the other hand, the highest power is achieved in setting 3, in which all active variables belong to the first block. Good power can also be observed in setting 5, where the majority of active variables is in the first block. As for the empirical FDR, it seems comparable across settings, varying in the range 0.18–0.26.

**Table 2:** Simulation study: description of simulation settings and the related performance

Setting	Parameters						penalizedclr		IPF-Lasso		clogitL1	
	$p_1$	$p_2$	$a_1$	$a_2$	$\beta _1$	$\beta _2$	Power	FDR	Power	FDR	Power	FDR
1	50	50	10	10	4	4	0.59	0.23	0.70	0.34	0.85	0.46
2	50	50	3	17	4	4	0.70	0.23	0.65	0.28		
3	50	50	20	0	4	0	0.84	0.18	1.00	0.69		
4	20	80	10	10	4	1	0.50	0.26	1.00	0.79	0.64	0.48
5	20	80	15	5	4	4	0.81	0.21	1.00	0.57		
6	20	80	5	15	4	4	0.58	0.21	0.90	0.68		

**Fig. 1:**
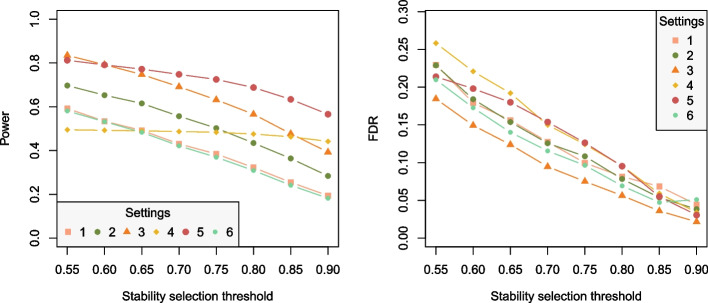
Empirical power and false discovery rate as a function of the threshold of the proposed variable selection procedure in 6 considered settings

We set the threshold for selection to 0.55, which is at the low end of the suggested range (Sect. "stable.clr.g function"). To evaluate the impact of this choice, we computed the empirical power and FDR for a grid of potential thresholds across the suggested range $(0.55 - 0.9)$. Results are shown in Fig. 1.

As expected, both power and FDR decrease with an increasing threshold for selection, since a stricter criterion for selection leads to fewer selected variables, both active and inactive. Ordering of the settings is largely preserved across different thresholds (with some exceptions, for instance, the power for settings 3 and 5). Interestingly, setting 4 stands out from the rest: while its FDR decreases with the increasing threshold, as expected, its power remains constant. Recall that in setting 4, the number of active variables is equal among the two blocks, but the signal strength in the second block is lower. Indeed, this signal seems to be too low to be picked up, and the variable selection procedure selects only the variables of the first block.

A related question is how the proposed method behaves with varying sample size. To investigate this issue, we considered setting 5 and generated 50 datasets of sample sizes 50, 100 and 500. Estimated power and FDR are shown in Table 3.

**Table 3:** Simulation study: performance for different sample sizes

Performance	Sample size			
	$n = 50$	$n = 100$	$n=200$	$n=500$
Power	0.08	0.44	0.81	0.97
FDR	0.07	0.11	0.21	0.22

We see that for the given signal strength (see Table 2) the method has no power for the smallest sample size. Already for $n=100$, nearly half of the active variables are identified. For $n=500$, the method almost always identifies all active variables. The estimated FDR remains around 0.2. When the sample size is large, and it is desirable to keep the number of false positives low, we might increase the threshold for selection. For instance, in our example, for $n=500$, by increasing the threshold to 0.95, the power decreases to 0.86 and FDR to 0.06.

The main purpose of the presented simulation study and the data application is to illustrate the possibilities and limitations of the proposed method. The comparison with IPF-Lasso and clogitL1, methods that take into account the block structure, but ignore matching, or vice versa, was reported, since, to the best of our knowledge, there are no other methods that implement penalized estimation of the conditional logistic regression model with multiple blocks of predictors.

The small simulation study has showed, in line with the reported results for the IPF-lasso [3], that taking into account the block structure of predictors brings an advantage when the blocks are indeed different, in terms of signal strength and/or the number or proportion of active variables. Otherwise, it is of course beneficial to treat all variables on equal standing, since in that case we are dealing with fewer tuning parameters. The comparison between conditional and unconditional penalized logistic models was also studied in [15]. Their results show that estimating conditional models when data come from a matched study is beneficial, especially when strata are large and the number of covariates is moderate.

### The NOWAC lung cancer dataset

To illustrate the proposed method in practice, we consider a lung cancer matched case–control study nested within the Norwegian Women and Cancer Study (NOWAC) [13], a prospective cohort study. Our data consist of 125 case–control pairs matched by time since blood sampling and year of birth, identified in the NOWAC cohort. Methylation levels and gene expression were measured in peripheral blood. We have focused on CpGs and genes that have previously been reported to be associated with smoking. In particular, we considered a list of CpGs differentially methylated between current smokers and nonsmokers according to [10]. Since the total number of reported CpGs, 18760, precludes us from including them all in a considered multivariate model, we have selected the top 5000 CpGs according to their reported $p$-values. After restricting attention to complete observations, we were left with 4370 CpGs. Similarly, we considered a list of differentially expressed genes between current smokers and nonsmokers reported in [9]. Here, of the 1270 reported genes, in NOWAC we have information on 943 which we included in our analysis.

Our goal was to select genes and CpGs that are associated with lung cancer status. Assuming a conditional logistic regression model, this amounts to selecting variables in the joint model that have a non-zero coefficient.

We started our analysis by searching for the data adaptive vector of penalty factors. We set the elastic net mixing parameter $\alpha =0.6$ and ran the function default.pf three times, since this function relies on cross validation for selecting the penalty in the tentative model producing results that may vary between runs. In our case, the average vector of penalty factors was proportional to (1, 3.6). We then ran find.default.lambda, to find $\lambda _1 = 5.3$.

For stability selection, we have considered the following list of penalty vectors: (5, 1), (5, 2), (5, 5), (5, 10), (5, 15), (5, 20). We intentionally included combinations of penalties that appear to be far from the estimated data adaptive penalty factor, both to allow for less overall penalization and to explore different relative penalties for the two blocks. Our motivation comes from the observation that when conducting stability selection, it is more desirable to err on the side of too little penalization than too much. In the former case, non-active variables are expected to vary randomly across different subsamples and achieve low selection probability. In the latter case, however, the large amount of penalization might negatively affect the power to identify active variables.

We set 0.55 as the threshold for selection, and ended up with selecting two CpGs: cg27039118 (estimated selection probability: 0.56), cg17065712 (0.56), and four genes: *MAPRE2* (0.63), *KCNMB1* (0.78), *ATP1B1* (0.61) and *SLC9A2* (0.6). Although they were all included in the analysis based on their reported association with smoking, none of these selected genes nor CpGs seem to have an established link to lung cancer.

### The TCGA lung adenocarcinoma dataset

We further illustrate our method on LUng Adenocarcinoma Dataset (LUAD) publicly available from TCGA. We downloaded the data from https://openml.org/search?type=data &status=any &id=42297, following the instructions in [7].

Data consist of survival times for 426 subjects diagnosed with lung adenocarcinoma. For each subject, data include information on a small number of clinical variables (age, sex, smoking history and cancer stage) as well as gene expression level (mRNA) and copy number variation (cnv).

To illustrate our method, we defined a binary response variable describing survival status at a three year mark (1 = alive, 0 = dead). Subjects that were censored prior to the three year mark were excluded from the main analysis.

This dataset does not come from a case control study, so for the purpose of the illustration, we matched subjects on the basis of the available clinical information. The 1:1 matching was exact on sex and based on the Mahalanobis distance for age and smoking history (for further details, we refer to the documentation of the R package MatchIt [8]. This left us with 65 case–control pairs.

Our goal was to identify, among measured features, those that are associated with the survival status three years from diagnosis. The total number of available mRNAs and copy number variations was more then 80000, so we performed initial filtering to select 1000 to include in the conditional logistic regression model. To this aim, we defined a binary variable having value 1 if the subject was diagnosed with stage III cancer and 0 otherwise (stages Ib and IIa). We then carried out two sample t-tests comparing mean levels of each feature in stage III group vs. others and selected those having lowest *p*-values. Among 1000 selected features, 802 were copy number variations and 198 were mRNAs. Filtering was performed on data not used in the main analysis, that is, on subjects not included in the 65 matched case - control pairs.

**Fig. 2:**
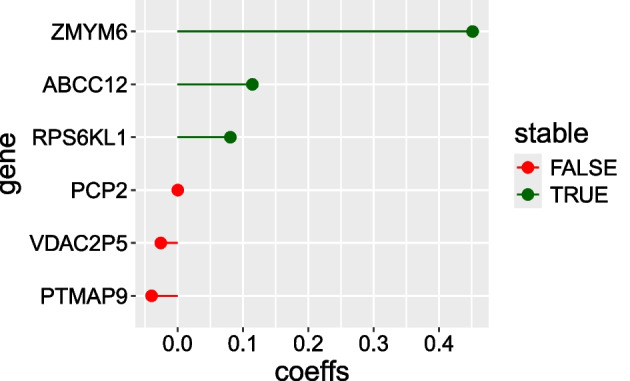
Non-zero estimated coefficients in the LUAD study. Those selected by stability selection are plotted in green

Our algorithm for data adaptive choice of penalty factors suggested excluding cnv from further analysis. For illustration purposes, we decided to keep them and considered an adhoc vector of penalty factors (4, 1) that corresponds to penalizing the cnv block 4 times as much as that of mRNA. For this vector of penalty factors, we found the optimal $\lambda = 9.37$. We thus fit a penalized conditional logistic regression model with the vector $\varvec{\lambda }=(40,10)^\top$ and $\alpha = 0.6$. This gave us 8 non-zero coefficients for the mRNA block, six of which are shown in Fig. 2. The remaining two correspond to novel genes, at the moment not annotated. No cnvs were selected.

**Fig. 3:**
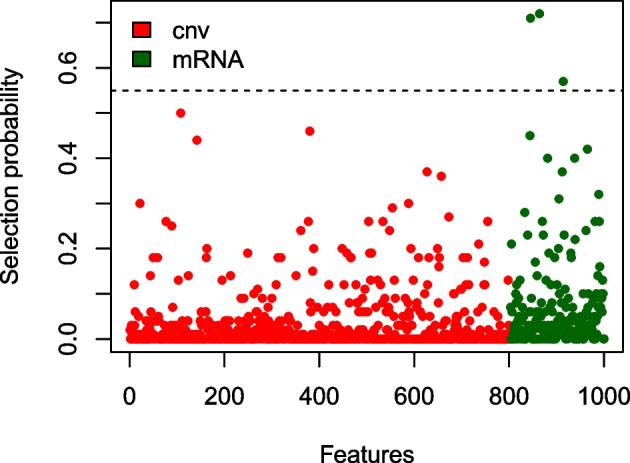
Selection probabilities for the considered features. The dashed line $y = 0.55$ represents the considered threshold for inclusion

We then performed stability selection with the list of penalty vectors: (7, 4), (15, 5), (4, 8), (2, 6). As in the previous example, we considered a wider range of penalty vectors to give variables of each block a chance to enter the model. For the choice $B=50$, the analysis took 26 s on a personal computer. Estimated selection probabilities are shown in Fig. 3. We see that setting the threshold at 0.55 leads to three stably selected features, all mRNAs also present in Fig. 2, shown in green. The importance of these features in the given context is, however, unclear.

## Conclusions

In this work we have presented our implementation of an algorithm that allows for fitting high dimensional conditional logistic regression models with covariates coming from different data sources. The output of the proposed method is a set of variables significantly associated with case–control status. To the best of our knowledge, no such software has so far been available in the statistical software R.

In the simulation study and the data application, we considered 1:1 matching, but the proposed method is suitable also for a general 1:*k* matching, for $k\ge 1$, where each case is matched to *k* controls.

In our implementation, we have opted for a data adaptive method for selecting penalty parameters that estimates tentative penalized model(s) and assigns less penalty to blocks that have higher mean estimated coefficients. Of course, there are many other sensible options for the choice of data adaptive penalty factors (see ipflasso R package). The user is free to combine the proposed estimation procedure with an arbitrary procedure for selecting penalty parameters.

We have implemented stability selection with the aim of stabilizing the obtained results in terms of selected variables. However, stability selection can also be used for Type 1 error control. In particular, [14] show how to bound the expected number of selected inactive variables by means of stability selection. Nevertheless, their method for ensuring error control relies on a nontrivial choice of tuning parameters, which is an interesting research question on its own. For this reason, we did not pursue this question in the present contribution.

## Availability and requirements

penalizedclr is implemented in R. Release versions are available on CRAN and work on all major operating systems. The development version is available at https://github.com/veradjordjilovic/penalizedclr.

**Project name:** penalizedclr R package

**Project home page:** https://CRAN.R-project.org/package=penalizedclr

**Operating system(s):** Platform independent.

**Programming language:** R

**Other requirements:** No.

**License:** MIT + file LICENSE

**Any restrictions to use by non-academics:** No.

## Data Availability

Data analyzed in Section "The NOWAC lung cancer dataset" cannot be shared publicly because of local and national ethical and security policies. Data access for researchers will be conditional on adherence to both the data access procedures of the NOWAC study and the UiT, The Arctic University of Norway (contact: Tonje Braaten tonje.braaten@uit.no) in addition to approval from the local ethical committee. Data analyzed in Section "The TCGA lung adenocarcinoma dataset" are publicly available.

## Appendix A: Technical details

### The conditional logistic regression model

We consider a binary outcome *Y* and its association with a vector of covariates ${\varvec{X}}$ grouped into *P* blocks: ${\varvec{X}}^1, {\varvec{X}}^2,\ldots , {\varvec{X}}^P$, where $P\ge 2$. We assume that available observations come from a matched case–control study, so that they are grouped into *n* clusters, each with one case and at least one control. We assume that the relationship between *Y* and ${\varvec{X}} = ({\varvec{X}}^1, \ldots , {\varvec{X}}^P)$ can be described by the conditional logistic regression model:

A1 $$\begin{aligned} \text {logit}\left[ {\textrm{P}(Y_{il}=1})\right] = \log \left[ \frac{\textrm{P}(Y_{il}=1)}{1- \textrm{P}(Y_{il}=1)}\right] = \beta _{0i} + {\varvec{X}}_{il}^\top \varvec{\beta }, \end{aligned}$$

where each observation is indexed by two indices: the first one indicating the cluster $i \in \left\{ 1,\ldots ,n\right\}$, and the second one indicating the observation within the cluster $l=1,\ldots ,K$, where *K* is the size of the cluster, common for all clusters.

### Estimation

The interest lies in estimating $\varvec{\beta }$, while cluster specific effects ${\beta }_{0i}$, $i=1,\ldots , n$ are treated as nuisance. If, without loss of generality, we assume that the index of a case is 1 within each cluster, the inference is based on the so-called conditional likelihood:

A2 $$\begin{aligned} L(\varvec{\beta }) = \prod _{i=1}^{n} L_i(\varvec{\beta })= \prod _{i=1}^{n}\frac{\exp \left\{ {\varvec{X}}_{i1}^\top \varvec{\beta }\right\} }{\sum _{l=1}^K\exp \left\{ {\varvec{X}}_{il}^\top \varvec{\beta }\right\} }, \end{aligned}$$

representing the likelihood conditional on there being exactly one case within each cluster.

Usually, the estimate of $\varvec{\beta }$ is obtained by maximizing the conditional log likelihood $\log L(\varvec{\beta })$. When the dimension of the parameter is high with respect to available sample size, this approach fails and the problem of estimation is commonly addressed by introducing a penalty term. The estimate of $\varvec{\beta }$ is then obtained by minimizing:

A3 $$\begin{aligned} -\log L(\varvec{\beta }) + \lambda \Vert \varvec{\beta }\Vert _1, \end{aligned}$$

where $\lambda >0$ is a tuning parameter, and the $L_1$ norm, denoted as $\Vert \varvec{\beta }\Vert _1$, represents a popular penalty choice.

In the presence of blocks of covariates, $\varvec{\beta }$ naturally partitions into subparamateres corresponding to the *P* blocks as $\varvec{\beta }= (\varvec{\beta }^{1}, \ldots , \varvec{\beta }^P)$. In our approach we allow for different penalites for different blocks, so that the estimate of $\varvec{\beta }$ is obtained by minimizing:

A4 $$\begin{aligned} -\log L(\varvec{\beta }) + \sum _{p=1}^P\lambda _p\Vert \varvec{\beta }^p\Vert _1. \end{aligned}$$

We also consider and implement the elastic net penalty so that the estimate of $\varvec{\beta }$ is obtained by minimizing:

A5 $$\begin{aligned} -\log L(\varvec{\beta }) + \sum _{p=1}^P\lambda _p\Vert \varvec{\beta }^p\Vert _1 + \frac{1-\alpha }{2\alpha }\sum _{p=1}^P\lambda _p\Vert \varvec{\beta }^p\Vert _2, \end{aligned}$$

where $\alpha \in \left( 0,1\right]$ is the so-called ”mixing” parameter. Note that this is a slightly different parametrization of the penalty term from the one analogous to the glmnet

$$\begin{aligned} \alpha \sum _{p=1}^P\lambda _p\Vert \varvec{\beta }^p\Vert _1 + \frac{1-\alpha }{2}\sum _{p=1}^P\lambda _p\Vert \varvec{\beta }^p\Vert _2. \end{aligned}$$

Note also that our algorithm is not able to estimate pure ridge models $\alpha =0$.

### Choice of the tuning parameters

Fitting the penalized conditional logistic regression model in (A5) requires setting $P+1$ parameters: the vector of penalties $\varvec{\lambda } = (\lambda _1, \ldots , \lambda _P)^\top$ and the elastic net parameter $\alpha$.

The elastic net parameter determines the balance between the $L_1$ and the $L_2$ penalty and is typically considered a higher order parameter that is either set apriori on subjective grounds or after experimenting with a few different values, see “An introduction to glmnet” https://glmnet.stanford.edu/articles/glmnet.html.

The vector $\varvec{\lambda } = (\lambda _1, \ldots , \lambda _P)^\top$ determines the level of penalization. In penalizedclr, the vector of penalties is parameterized as the product of the scalar $\lambda$, determining the overall level of penalization and the vector of penalty factors: $(1, \ldots , \lambda _P/\lambda )^\top$, determining the relative penalization for different blocks.

The scalar parameter is determined by cross-validation as follows. A subset of strata is left out at random. Without loss of generality, we assume that strata indexed $1, \ldots , m$ are left out. A penalized conditional logistic regression model is fit to the non left out strata for a sequence of $\lambda$. For each $\lambda$, the cross validated log conditional likelihood is computed as

A6 $$\begin{aligned} CV(\lambda ) = \log L_{(m)}(\hat{\varvec{\beta }}_{-(m)}), \end{aligned}$$

where $\log L_{(m)}$ is the conditional log likelihood computed on strata $1,\ldots ,m$, and $\hat{\varvec{\beta }}_{-(m)}$ is the estimate of $\varvec{\beta }$ obtained by minimizing the penalized conditional log likelihood in (A5) on data excluding strata $1,\ldots ,m$. The optimal $\lambda$ maximizes (A6). In our implementation, we choose the value of $\lambda$ following the “1 standard error” rule: we choose $\lambda$ that selects the simplest model with estimated $CV(\lambda )$ within 1 standard deviation of the minimum *CV*.

The vector of penalty factors is chosen in a data adaptive fashion by a heuristic strategy described in Section "Data adaptive choice of penalty parameters". Note that we set relative penalties inversely proportional to the mean of estimated coefficients from a tentative conditional logistic model in (A3), however different functions of estimated coefficients could be used instead. For instance, an empirical study in [16] compares the performance of the method based on the arithmetic and geometric means.

A related question is how to specify the set of penalty vectors when performing stability selection. Our advice is to use the vector found by the data adaptive strategy and cross validation as a starting point, and to include vectors with lower levels of overall penalization as well as those with different vectors of penalty factors. In this way, the power to select important variables should increase, and variables of all blocks are given a chance to enter the model. We refer to examples in Sections "The NOWAC lung cancer dataset" and "The TCGA lung adenocarcinoma dataset" for illustration.
